# Supplementary material for: Neural network conditioned to produce thermophilic protein sequences can increase thermal stability
Source: Sci Rep. 2025 Apr 23;15:14124. doi: 10.1038/s41598-025-90828-0 (PMC12019596; doi:10.1038/s41598-025-90828-0)
Supplement: Supplementary file 1 — Supplementary Information. [file 41598_2025_90828_MOESM1_ESM.pdf]

Supplementary Information for "*Neural network conditioned to produce thermophilic protein sequences can increase thermal stability*"

Dataset used

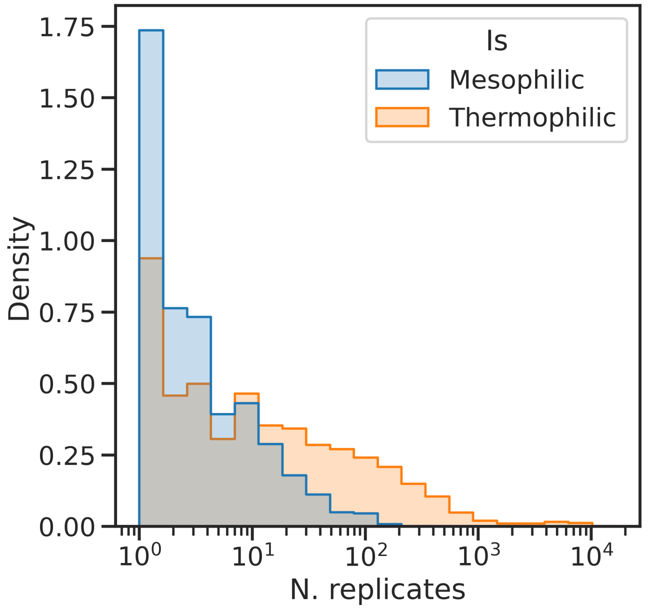

Figure S1: Density of replicate counts for mesophilic and thermophilic sequences occurring in the training set.[1] Orange is the number of mesophilic counterparts per thermophile, and blue vice versa. The most common number of replicates for both the input and the target sequences is one, however the majority of proteins occur multiple times (paired to a different homolog) in the training set. Thermophilic replicates are right skewed, as homologous protein pairs were identified for a much larger group of mesophilic sequences to a smaller set of thermophilic ones.

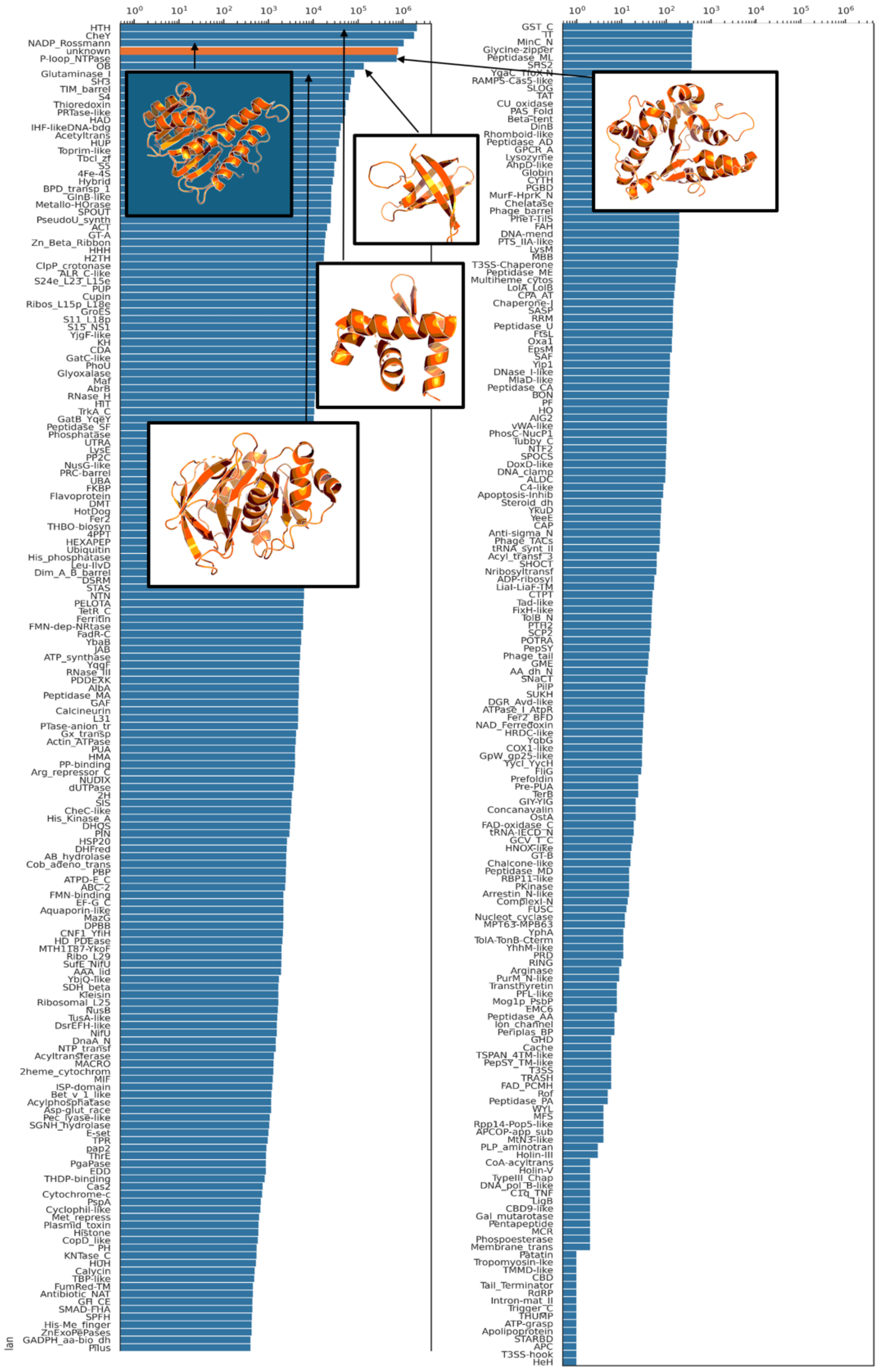

Figure S2: Counts of Pfam clans observed in the dataset for NOMELT, with structures for some of the highest represented shown (HTH, NADP_Rossmann, P-Loop NTPase, OB, Glutaminase 1)[2]

Table S1: Counts of each Pfam IDs observed for meso sequences in the dataset.[2]

ID	count	ID	count	ID	count	ID	count
**PF00578.24**	10518	PF17954.4	156	PF00301.23	1834	PF18931.3	4
**PF08534.13**	10442	PF20393.1	15	PF02075.20	1068	PF03367.16	6
**PF10417.12**	870	PF14065.9	3	PF03652.18	4955	PF01063.22	5
**PF04463.15**	650	PF02517.19	60	PF00575.26	1193	PF11699.11	35
**PF01257.22**	619	PF17144.7	35	PF13275.9	721	PF05952.15	5
**PF02834.19**	1511	PF00444.21	9688	PF01259.21	3764	PF07510.14	21
**PF13563.9**	1540	PF08069.15	31	PF00269.23	999	PF02680.17	22
**PF10469.12**	355	PF01649.21	7486	PF01127.25	458	PF09685.13	52
**PF00106.28**	310642	PF06026.17	637	PF00584.23	230	PF08849.14	6
**PF01073.22**	549	PF01192.25	331	PF04472.15	433	PF18480.4	5
**PF01370.24**	45633	PF02036.20	43	PF03749.16	908	PF13738.9	11
**PF02719.18**	560	PF02775.24	817	PF17746.4	908	PF06763.14	8
**PF07993.15**	16	PF02814.18	39	PF07638.14	2006	PF01876.19	28
**PF08659.13**	255649	PF05194.15	31	PF04539.19	790	PF10135.12	8
**PF13561.9**	310649	PF04893.20	49	PF04402.17	116	PF09701.13	4
**PF16363.8**	934	PF14164.9	10	PF02146.20	1221	PF09912.12	6
**PF02829.17**	1393	PF14256.9	61	PF01380.25	221	PF10730.12	32
**PF08279.15**	4445	PF00882.21	30	PF13580.9	1033	PF03745.17	13
**PF03061.25**	1152	PF02737.21	122	PF01464.23	243	PF11706.11	5
**PF14539.9**	68	PF13622.9	32	PF02616.17	1682	PF05707.15	9
**PF13279.9**	710	PF17837.4	1043	PF01668.21	4449	PF14516.9	1
**PF01812.23**	1604	PF00975.23	39	PF01713.24	127	PF04647.18	11
**PF00005.30**	454734	PF08386.13	11	PF09335.14	3155	PF01510.28	50
**PF02463.22**	96155	PF12746.10	65	PF00081.25	5677	PF08979.14	5
**PF13175.9**	812	PF13480.10	61	PF02777.21	5695	PF16277.8	2
**PF13476.9**	4126	PF04326.17	4	PF06686.14	3156	PF04967.15	5
**PF13191.9**	9197	PF05219.15	33	PF12685.10	156	PF06253.14	1
**PF13304.9**	65167	PF00329.22	20	PF12116.11	416	PF03824.19	7
**PF00406.25**	12309	PF04677.18	75	PF01944.20	291	PF01311.23	4
**PF05191.17**	11095	PF04290.15	181	PF09548.13	1935	PF07758.14	11
**PF13207.9**	12659	PF01487.18	22	PF09580.13	61	PF13803.9	3

**PF13238.9**	7047	PF04342.15	13	PF09579.13	156	PF18910.3	14
**PF13671.9**	3819	PF13743.9	37	PF14034.9	125	PF20548.1	9
**PF01202.25**	1765	PF07610.14	15	PF02590.20	6738	PF04068.18	19
**PF00485.21**	1490	PF09891.12	7	PF00588.22	10681	PF06855.15	55
**PF02224.21**	7927	PF10946.11	15	PF08032.15	3144	PF01637.21	20
**PF13189.9**	4901	PF10958.11	15	PF13782.9	124	PF10882.11	1
**PF12399.11**	22294	PF04214.16	1	PF03862.16	3627	PF08269.14	3
**PF01656.26**	500	PF14076.9	4	PF14097.9	104	PF17200.7	3
**PF13500.9**	232	PF14084.9	91	PF04026.15	422	PF07027.15	3
**PF02223.20**	4315	PF04322.15	17	PF04232.15	4797	PF01629.19	7
**PF13521.9**	1965	PF01949.19	17	PF02586.17	1103	PF10704.12	27
**PF13555.9**	4915	PF03372.26	122	PF00436.28	2604	PF09985.12	1
**PF13604.9**	450	PF02943.18	21	PF08141.15	383	PF18933.3	6
**PF00142.21**	44	PF10646.12	56	PF14098.9	193	PF12788.10	1
**PF02374.18**	33	PF13506.9	10	PF01740.24	3214	PF09824.12	12
**PF06564.15**	42	PF07449.14	74	PF13466.9	3127	PF04229.17	14
**PF09140.14**	127	PF11209.11	6	PF01300.21	562	PF07747.14	1
**PF10609.12**	240	PF04390.15	37	PF04143.17	66	PF04207.15	11
**PF13614.9**	268	PF03956.16	28	PF01975.20	172	PF05845.15	10
**PF00696.31**	10687	PF05430.14	42	PF00923.22	8543	PF02697.17	1
**PF01061.27**	556	PF09754.12	17	PF01925.22	406	PF17334.5	20
**PF12679.10**	396	PF13263.9	20	PF01361.24	718	PF04341.15	7
**PF06182.14**	7	PF05036.16	13	PF14552.9	458	PF00459.28	2
**PF09819.12**	118	PF01841.22	23	PF07331.14	133	PF06713.14	6
**PF03215.18**	224	PF03686.16	5	PF03741.19	419	PF05400.16	2
**PF08352.15**	163	PF14043.9	19	PF00440.26	7901	PF08734.14	15
**PF00583.28**	10471	PF14068.9	92	PF16859.8	284	PF03672.16	36
**PF08445.13**	2181	PF02591.18	156	PF08361.14	32	PF14140.9	8
**PF13302.10**	6899	PF00910.25	40	PF17932.4	2820	PF13115.9	13
**PF13420.10**	4842	PF12568.11	3	PF17938.4	239	PF09873.12	5
**PF13508.10**	6490	PF01989.19	37	PF08359.14	798	PF11906.11	1
**PF13673.10**	6310	PF00165.26	88	PF08362.14	15	PF14340.9	29
**PF13527.10**	573	PF02805.19	13	PF13977.9	392	PF19788.2	4
**PF13523.9**	1218	PF12833.10	94	PF01910.20	1981	PF03928.17	19
**PF00694.22**	3499	PF00025.24	78	PF07615.14	11	PF04140.17	41
**PF01648.23**	6505	PF16554.8	67	PF00899.24	679	PF04191.16	36
**PF01842.28**	7840	PF07565.16	21	PF13899.9	271	PF00497.23	20
**PF10369.12**	4300	PF01145.28	3	PF02597.23	6069	PF10613.12	17
**PF13291.9**	2355	PF02617.20	30	PF06738.15	433	PF03787.18	5

**PF13710.9**	4188	PF01983.19	35	PF12821.10	461	PF02676.17	9
**PF03315.18**	1602	PF19769.2	12	PF00121.21	1347	PF00775.24	1
**PF00571.31**	4349	PF14358.9	29	PF00265.21	651	PF08551.13	4
**PF13740.9**	1132	PF01578.23	153	PF02581.20	1548	PF04016.15	32
**PF00708.21**	1161	PF07311.15	208	PF04263.19	2050	PF14126.9	23
**PF01553.24**	1329	PF07187.14	18	PF04265.17	1941	PF13746.9	2
**PF03352.16**	664	PF07423.14	16	PF00590.23	2396	PF20028.2	3
**PF04008.17**	80	PF11387.11	48	PF01709.23	12135	PF06056.15	8
**PF03435.21**	960	PF11151.11	24	PF01980.19	88	PF02926.20	1
**PF13460.9**	12353	PF11167.11	35	PF04073.18	990	PF18489.4	6
**PF04321.20**	483	PF13056.9	9	PF01746.24	3727	PF06938.14	7
**PF05988.15**	106	PF04242.16	8	PF02583.20	1902	PF10694.12	11
**PF00085.23**	10352	PF14359.9	12	PF01371.22	778	PF13938.9	6
**PF13098.9**	7590	PF20020.2	5	PF01509.21	6	PF19651.2	7
**PF13905.9**	3610	PF20567.1	5	PF16198.8	6	PF13579.9	4
**PF13728.9**	462	PF03151.19	3	PF00814.28	4065	PF02190.19	10
**PF14595.9**	2267	PF01269.20	37	PF02367.20	1139	PF01969.20	8
**PF01987.20**	188	PF19579.2	5	PF01206.20	1536	PF11964.11	1
**PF01168.23**	8724	PF16784.8	10	PF02580.19	15754	PF13400.9	1
**PF01081.22**	2611	PF12645.10	97	PF03167.22	1116	PF14182.9	1
**PF00596.24**	3604	PF00475.21	13206	PF03649.16	102	PF07947.17	8
**PF00072.27**	17988<br>28	PF02505.17	14	PF01894.20	3529	PF02594.19	19
**PF03861.17**	812	PF05521.14	69	PF03653.16	319	PF06790.14	2
**PF01583.23**	399	PF07929.14	40	PF02021.20	1503	PF04101.19	12
**PF01261.27**	158	PF01948.21	141	PF04297.17	547	PF02996.20	4
**PF03079.17**	69	PF02748.18	141	PF03698.16	262	PF03911.19	6
**PF01316.24**	3874	PF06271.15	57	PF00547.21	892	PF16083.8	3
**PF02863.21**	3850	PF03876.20	39	PF01730.19	328	PF19620.2	11
**PF00011.24**	1398	PF01172.21	47	PF00582.29	12057	PF13040.9	6
**PF17886.4**	1188	PF09377.13	47	PF02151.22	1945	PF00361.23	3
**PF01037.24**	5224	PF20268.1	47	PF06257.14	205	PF01920.23	20
**PF13404.9**	3672	PF10747.12	20	PF02706.18	518	PF19785.2	18
**PF13412.9**	4993	PF01922.20	6	PF01797.19	533	PF13768.9	1
**PF01047.25**	2124	PF02657.18	44	PF07873.14	726	PF06339.15	19
**PF09339.13**	1472	PF03592.19	29	PF02575.19	5393	PF07279.14	2
**PF12802.10**	3569	PF05256.15	29	PF02130.20	1793	PF10027.12	11
**PF12840.10**	2199	PF01050.21	25	PF01906.20	1736	PF11042.11	8
**PF03780.16**	3473	PF01476.23	190	PF02620.20	1334	PF08882.14	9

**PF02261.19**	1733	PF05425.16	101	PF04264.16	447	PF09902.12	35
**PF01177.25**	1154	PF08905.14	7	PF03795.17	326	PF11255.11	6
**PF03477.19**	4638	PF09413.13	22	PF02542.19	6668	PF04009.15	6
**PF00119.23**	927	PF10006.12	53	PF02325.20	115	PF04609.15	8
**PF00430.21**	1040	PF12758.10	23	PF01809.21	875	PF05440.15	4
**PF00137.24**	711	PF04238.15	169	PF11068.11	67	PF04985.17	7
**PF00401.23**	2436	PF14947.9	69	PF04296.16	445	PF13453.9	8
**PF02823.19**	2800	PF04356.15	20	PF14162.9	22	PF00173.31	10
**PF02310.22**	2379	PF00478.28	39	PF09424.13	10140	PF18899.3	5
**PF02607.20**	1405	PF13240.9	69	PF14166.9	32	PF14385.9	19
**PF00216.24**	26123	PF04930.18	4	PF14115.9	7	PF04208.17	15
**PF18291.4**	14524	PF04186.16	133	PF05164.16	89	PF01877.20	9
**PF01313.22**	3152	PF04982.16	13	PF01258.20	434	PF12800.10	11
**PF02397.19**	192	PF13551.9	217	PF02535.25	574	PF06434.16	1
**PF02577.17**	772	PF07071.14	8	PF09723.13	257	PF08679.14	4
**PF00364.25**	3419	PF00384.25	42	PF04298.15	2261	PF04340.15	13
**PF13533.9**	1028	PF01568.24	14	PF01712.22	500	PF01886.19	4
**PF02632.17**	777	PF00900.23	39	PF06414.15	45	PF12773.10	35
**PF12822.10**	1293	PF08071.15	39	PF13401.9	1822	PF05729.15	2
**PF01722.21**	74	PF05433.18	37	PF03379.16	366	PF11307.11	8
**PF00528.25**	25647	PF08501.14	39	PF00326.24	225	PF10764.12	8
**PF03099.22**	32	PF09345.13	11	PF00561.23	474	PF07437.14	7
**PF02559.19**	5476	PF04386.16	133	PF05728.15	6	PF06342.15	1
**PF02570.18**	2260	PF07811.15	48	PF12146.11	811	PF02796.18	16
**PF02571.17**	145	PF19344.2	42	PF12697.10	554	PF00015.24	4
**PF08220.15**	1455	PF14246.9	94	PF19304.2	88	PF13340.9	9
**PF02754.19**	1408	PF13414.9	62	PF13840.9	64	PF14367.9	11
**PF02457.19**	9	PF04892.15	36	PF08643.13	1014	PF03413.22	16
**PF19293.2**	8	PF13519.9	48	PF08327.14	266	PF02086.18	8
**PF06153.14**	3824	PF03853.18	15	PF00731.23	3042	PF13635.9	1
**PF01066.24**	4670	PF09643.13	35	PF01918.24	38	PF20116.2	4
**PF04509.15**	2253	PF03899.18	33	PF01520.21	421	PF18423.4	13
**PF13690.9**	1027	PF12431.11	87	PF02558.19	102	PF07385.15	4
**PF03975.16**	2117	PF13668.9	13	PF03869.17	2	PF01939.19	7
**PF01584.22**	1499	PF08123.16	16	PF01951.19	92	PF14177.9	15
**PF02417.18**	1905	PF01982.19	22	PF03960.18	1102	PF12654.10	13
**PF01467.29**	18575	PF08859.14	1	PF00733.24	564	PF04289.15	8
**PF08218.14**	3319	PF09626.13	1	PF01990.20	255	PF04900.15	8
**PF00574.26**	16752	PF09885.12	19	PF01171.23	192	PF18477.4	12

**PF02353.23**	1147	PF09922.12	64	PF05437.15	217	PF03929.19	4
**PF08241.15**	6539	PF10029.12	2	PF03483.20	198	PF16357.8	2
**PF13489.9**	5765	PF10047.12	8	PF04519.16	99	PF03872.16	4
**PF13649.9**	10142	PF10988.11	9	PF00936.22	1197	PF11553.11	11
**PF01209.21**	4761	PF14276.9	44	PF04972.20	116	PF05973.17	9
**PF08242.15**	5467	PF19803.2	37	PF09704.13	99	PF14122.9	11
**PF13847.9**	12546	PF14382.9	8	PF01881.19	176	PF01757.25	4
**PF05175.17**	7867	PF00736.22	36	PF18011.4	143	PF09919.12	6
**PF08003.14**	238	PF10662.12	18	PF00125.27	159	PF00218.24	3
**PF07736.14**	1894	PF04723.17	255	PF00808.26	174	PF15601.9	3
**PF00027.32**	9575	PF07739.16	9	PF15511.9	120	PF06028.14	3
**PF13545.9**	9809	PF08712.14	19	PF01955.21	8	PF20434.1	8
**PF00325.23**	1394	PF05105.15	30	PF03918.17	88	PF09871.12	6
**PF01121.23**	4919	PF13797.9	5	PF13380.9	474	PF18978.3	8
**PF07875.15**	137	PF01981.19	47	PF02674.19	350	PF06995.14	4
**PF02629.22**	2831	PF20161.2	20	PF06133.14	590	PF05406.18	2
**PF06971.16**	2757	PF00428.22	50	PF04378.16	234	PF09838.12	24
**PF01144.26**	1611	PF02556.17	45	PF00116.23	214	PF13780.9	4
**PF02572.18**	2033	PF07070.14	15	PF02348.22	699	PF01832.23	2
**PF02654.18**	644	PF09578.13	61	PF13443.9	1321	PF12852.10	1
**PF02283.19**	3323	PF05119.15	31	PF03091.18	317	PF16155.8	9
**PF02492.22**	702	PF00092.31	53	PF02560.17	51	PF19776.2	5
**PF01923.21**	2496	PF02625.19	11	PF05103.16	260	PF01900.22	4
**PF10719.12**	169	PF02618.19	35	PF04657.16	353	PF01078.24	15
**PF03602.18**	6862	PF14120.9	53	PF00226.34	145	PF00205.25	2
**PF06325.16**	1593	PF10116.12	17	PF04364.16	120	PF02552.19	5
**PF02475.19**	623	PF18856.4	17	PF01323.23	832	PF13709.9	23
**PF09445.13**	1255	PF14487.9	36	PF06949.14	119	PF13858.9	6
**PF01170.21**	164	PF07315.14	18	PF07098.14	12	PF04965.17	1
**PF05971.15**	183	PF09946.12	3	PF07457.14	57	PF00586.27	9
**PF12652.10**	209	PF13785.9	1	PF07561.14	84	PF03796.18	9
**PF00510.21**	790	PF20202.1	4	PF07892.14	590	PF09897.12	8
**PF00166.24**	15021	PF13510.9	42	PF08818.14	360	PF10050.12	6
**PF02537.18**	3820	PF06923.14	2	PF13376.9	197	PF12459.11	10
**PF09827.12**	722	PF13184.9	30	PF08830.13	117	PF17326.5	7
**PF01985.24**	1580	PF04039.16	21	PF08984.14	274	PF17910.4	1
**PF00313.25**	29375	PF00114.22	97	PF02641.18	106	PF14493.9	3
**PF02599.19**	294	PF03309.17	30	PF08924.14	5	PF15985.8	3
**PF05848.14**	2318	PF04608.16	146	PF09218.13	4	PF01427.20	1

**PF17727.4**	2288	PF10262.12	53	PF09393.13	88	PF04071.15	5
**PF02682.19**	217	PF17918.4	37	PF09917.12	18	PF04999.16	10
**PF01381.25**	4749	PF06265.14	62	PF09953.12	46	PF01052.23	5
**PF07883.14**	3461	PF17032.8	103	PF10105.12	309	PF01907.22	7
**PF12844.10**	3856	PF13479.9	29	PF10751.12	25	PF14178.9	3
**PF13560.9**	3677	PF04376.16	31	PF10934.11	28	PF03400.16	2
**PF13744.9**	67	PF04377.18	31	PF11208.11	46	PF12986.10	15
**PF00529.23**	80	PF03703.17	90	PF11953.11	271	PF02077.18	4
**PF13437.9**	342	PF10133.12	98	PF04025.15	812	PF03729.16	7
**PF16576.8**	339	PF07609.14	10	PF12670.10	90	PF18935.3	2
**PF03932.17**	91	PF08838.13	36	PF12953.10	114	PF01210.26	11
**PF04199.16**	2862	PF08922.14	11	PF13155.9	23	PF09650.13	4
**PF00033.22**	410	PF10752.12	14	PF13511.9	21	PF01780.22	8
**PF01292.23**	279	PF10827.11	15	PF14114.9	35	PF01935.20	1
**PF13631.9**	412	PF11848.11	28	PF01588.23	686	PF06847.14	3
**PF13442.9**	357	PF12227.11	31	PF14794.9	200	PF13559.9	3
**PF01219.22**	549	PF12438.11	8	PF04343.16	156	PF18823.4	1
**PF01569.24**	508	PF19573.2	13	PF04359.17	101	PF16955.8	1
**PF02734.20**	1008	PF04919.15	13	PF04634.15	16	PF11155.11	6
**PF01113.23**	421	PF02108.19	19	PF20099.2	5	PF05908.14	3
**PF05173.17**	405	PF10294.12	63	PF20114.2	26	PF14497.9	4
**PF00132.27**	4954	PF04350.16	41	PF20353.1	24	PF03685.16	9
**PF08503.13**	1328	PF04247.15	76	PF01904.21	246	PF20068.2	1
**PF14602.9**	2389	PF00080.23	83	PF06041.14	69	PF03668.18	1
**PF00692.22**	3166	PF08179.15	17	PF06103.14	13	PF00302.21	2
**PF06559.14**	470	PF02342.21	19	PF00563.23	8	PF10819.11	2
**PF00383.26**	6971	PF00515.31	60	PF00892.23	484	PF20221.1	5
**PF08211.15**	2862	PF13181.9	8	PF09512.13	74	PF05066.16	10
**PF14437.9**	4035	PF13424.9	54	PF12787.10	3	PF01870.21	8
**PF01230.26**	5456	PF13432.9	60	PF10576.12	214	PF14369.9	5
**PF11969.11**	5336	PF14559.9	61	PF03319.16	488	PF06754.15	7
**PF13358.9**	33	PF05753.17	1	PF09439.13	29	PF18552.4	7
**PF01791.12**	5731	PF14168.9	52	PF10955.11	126	PF03744.16	6
**PF00455.25**	607	PF19842.2	13	PF12682.10	84	PF10869.11	3
**PF01880.21**	484	PF12841.10	137	PF12724.10	271	PF17881.4	24
**PF06397.15**	344	PF02129.21	19	PF03963.17	177	PF05370.14	4
**PF00186.22**	1141	PF00837.20	8	PF13860.9	127	PF07878.14	3
**PF01872.20**	1445	PF01808.21	45	PF13861.9	119	PF01997.19	4
**PF10418.12**	298	PF12895.10	20	PF04316.16	146	PF03852.18	6

**PF01220.22**	3798	PF04234.15	14	PF02049.21	408	PF02924.17	2
**PF05163.15**	114	PF03626.17	48	PF10676.12	265	PF05014.18	1
**PF12867.10**	151	PF01266.27	52	PF02527.18	2830	PF07790.14	3
**PF06491.14**	459	PF02593.17	23	PF13704.9	95	PF08769.14	9
**PF01478.21**	251	PF01472.23	25	PF18029.4	925	PF00990.24	4
**PF06750.16**	224	PF09183.13	18	PF03458.16	219	PF02085.19	15
**PF01965.27**	10910	PF17832.4	2	PF12700.10	32	PF08732.13	9
**PF00117.31**	28315	PF14128.9	20	PF01288.23	835	PF10067.12	3
**PF07685.17**	17321	PF07840.15	9	PF08348.14	320	PF13632.9	4
**PF13507.9**	7447	PF03646.18	48	PF13309.9	320	PF11738.11	2
**PF00885.22**	4950	PF19581.2	15	PF01614.21	592	PF13739.9	2
**PF01035.23**	940	PF14089.9	61	PF04279.18	93	PF13349.9	1
**PF02635.18**	874	PF02405.19	40	PF01695.20	141	PF04457.15	6
**PF13686.9**	557	PF01077.25	97	PF18306.4	144	PF02548.18	7
**PF02683.18**	1835	PF03460.20	91	PF03968.17	12	PF14439.9	16
**PF13386.9**	956	PF03013.17	40	PF20293.1	4	PF01565.26	6
**PF04358.16**	624	PF00827.20	39	PF09278.14	266	PF00805.25	1
**PF00035.29**	2369	PF13333.9	44	PF05401.14	735	PF13599.9	1
**PF00636.29**	2561	PF13683.9	50	PF01795.22	184	PF16079.8	2
**PF14622.9**	2369	PF06093.16	33	PF04013.15	24	PF03096.17	7
**PF00908.20**	1198	PF17928.4	5	PF02470.23	120	PF09999.12	5
**PF06935.14**	51	PF17934.4	77	PF20560.1	91	PF14192.9	1
**PF07235.14**	48	PF01938.23	11	PF10057.12	264	PF19991.2	6
**PF02576.20**	3416	PF02830.21	7	PF04093.15	180	PF09243.13	3
**PF17384.5**	3388	PF09648.13	29	PF13241.9	561	PF17146.7	8
**PF07872.14**	229	PF06052.15	10	PF14824.9	71	PF07485.14	9
**PF02622.18**	482	PF16561.8	4	PF02540.20	324	PF11417.11	1
**PF08827.14**	173	PF02498.20	11	PF02665.17	131	PF20408.1	3
**PF09148.13**	476	PF03374.17	10	PF14512.9	1145	PF08973.13	7
**PF01205.22**	2147	PF06677.15	3	PF00877.22	91	PF12640.10	5
**PF09186.14**	2084	PF08819.14	36	PF01471.21	224	PF03604.16	1
**PF09862.12**	210	PF02643.18	23	PF07963.15	428	PF02954.22	10
**PF10673.12**	81	PF04325.16	27	PF08334.14	151	PF05618.14	5
**PF11084.11**	81	PF17259.5	33	PF04756.16	124	PF06676.14	4
**PF10966.11**	45	PF05187.16	57	PF01058.25	307	PF01595.23	2
**PF11146.11**	501	PF09383.13	39	PF03551.17	767	PF14208.9	6
**PF11148.11**	506	PF12801.10	23	PF03740.16	468	PF02634.18	3
**PF11213.11**	126	PF02637.21	163	PF02163.25	232	PF03544.17	10
**PF03625.17**	650	PF05768.17	58	PF01850.24	660	PF09749.12	6

**PF11588.11**	200	PF07754.14	21	PF02887.19	116	PF04488.18	5
**PF03885.16**	152	PF01849.21	26	PF02666.18	290	PF05704.15	5
**PF04167.16**	323	PF19026.3	23	PF01329.22	192	PF09997.12	13
**PF01751.25**	15229	PF06243.14	12	PF01862.19	41	PF10737.12	11
**PF13331.9**	995	PF00833.21	11	PF02565.18	1227	PF07826.14	12
**PF04239.15**	1602	PF09851.12	62	PF11967.11	1229	PF11085.11	4
**PF14277.9**	193	PF06695.14	26	PF13114.9	356	PF02985.25	2
**PF04284.16**	502	PF00565.20	19	PF00581.23	879	PF04368.16	6
**PF04361.16**	105	PF13848.9	5	PF00825.21	945	PF19451.2	3
**PF04430.17**	114	PF17099.8	85	PF01196.22	3863	PF09869.12	11
**PF04456.15**	788	PF02694.18	154	PF00276.23	4621	PF14329.9	3
**PF04461.16**	1499	PF04336.15	77	PF00076.25	130	PF16694.8	4
**PF17248.5**	36	PF00070.30	2	PF13165.9	392	PF14335.9	3
**PF04474.15**	1597	PF00670.24	1	PF11823.11	76	PF00457.20	21
**PF18917.3**	54	PF14173.9	3	PF01488.23	57	PF03657.16	4
**PF20074.2**	240	PF04978.15	24	PF13501.9	62	PF17833.4	4
**PF20111.2**	79	PF09835.12	99	PF10957.11	17	PF09945.12	5
**PF20140.2**	24	PF09920.12	1	PF09581.13	245	PF19567.2	5
**PF05949.15**	1314	PF10096.12	14	PF09560.13	80	PF13564.9	3
**PF05979.15**	75	PF11118.11	77	PF12164.11	64	PF12686.10	7
**PF00156.30**	37612	PF11290.11	21	PF00902.21	409	PF04254.16	14
**PF18912.3**	612	PF03994.17	115	PF16925.8	141	PF00512.28	9
**PF07155.15**	586	PF13799.9	13	PF17940.4	63	PF14343.9	9
**PF09515.13**	104	PF06475.14	28	PF13305.9	62	PF13972.9	6
**PF00378.23**	18	PF10518.12	21	PF08360.14	156	PF17194.7	1
**PF16113.8**	17	PF04468.15	2	PF06283.14	338	PF06304.14	2
**PF01132.23**	19222	PF00318.23	2003	PF02511.18	110	PF19094.3	8
**PF08207.15**	19223	PF16999.8	8	PF05717.16	107	PF12723.10	3
**PF09285.14**	19222	PF09957.12	37	PF05724.14	132	PF16906.8	24
**PF01176.22**	17826	PF02592.18	10	PF04226.16	202	PF13883.9	1
**PF03608.16**	116	PF13429.9	9	PF01169.22	359	PF04467.15	5
**PF03610.19**	884	PF17874.4	3	PF00699.23	250	PF08332.13	3
**PF04493.17**	71	PF18175.4	952	PF01991.21	198	PF13474.9	3
**PF01012.24**	438	PF02553.18	28	PF06156.16	299	PF02291.18	3
**PF07110.14**	230	PF08617.13	10	PF14070.9	36	PF13173.9	4
**PF01878.21**	400	PF03308.19	30	PF14035.9	266	PF02900.21	2
**PF02609.19**	112	PF04038.15	9	PF08741.13	155	PF14682.9	7
**PF07977.16**	4798	PF02603.19	15	PF14116.9	182	PF14689.9	7
**PF00890.27**	59	PF01984.23	37	PF13451.9	200	PF09964.12	5

**PF01494.22**	38	PF09886.12	7	PF04029.17	272	PF17341.5	2
**PF03486.17**	26	PF02698.20	42	PF05635.14	33	PF03934.16	2
**PF07992.17**	104	PF11877.11	26	PF03475.17	83	PF10741.12	3
**PF12831.10**	38	PF04070.15	82	PF04476.16	41	PF13689.9	6
**PF00392.24**	18755	PF13548.9	14	PF02096.23	109	PF16123.8	1
**PF07729.15**	5389	PF04475.15	16	PF12698.10	544	PF14152.9	3
**PF01521.23**	1691	PF05598.14	13	PF13338.9	43	PF19824.2	13
**PF04023.17**	645	PF13492.9	33	PF12689.10	9	PF02687.24	6
**PF01926.26**	2090	PF02893.23	3	PF02675.18	415	PF06189.15	2
**PF02421.21**	1934	PF09585.13	15	PF02311.22	248	PF04969.19	15
**PF03193.19**	272	PF12535.11	37	PF01813.20	384	PF11716.11	2
**PF00111.30**	2756	PF06841.15	18	PF03591.17	340	PF01398.24	1
**PF01799.23**	3731	PF03334.17	13	PF08840.14	100	PF00356.24	4
**PF13085.9**	997	PF01090.22	39	PF01312.22	83	PF00532.24	4
**PF13183.9**	897	PF14601.9	3	PF01152.24	209	PF13407.9	2
**PF13237.9**	2563	PF09346.13	12	PF13375.9	43	PF06347.16	4
**PF13534.9**	699	PF14567.9	9	PF08000.14	260	PF09858.12	1
**PF00037.30**	3419	PF14568.9	12	PF14470.9	190	PF18950.3	7
**PF12797.10**	586	PF08770.14	17	PF14526.9	16	PF19965.2	1
**PF12837.10**	1576	PF12638.10	20	PF01930.20	698	PF08863.13	3
**PF12838.10**	2951	PF01893.19	6	PF12705.10	55	PF14101.9	3
**PF13187.9**	2025	PF03959.16	26	PF01891.19	416	PF16661.8	3
**PF14697.9**	1328	PF08798.14	78	PF08666.15	89	PF01000.29	1
**PF13247.9**	835	PF01976.20	7	PF13144.9	21	PF17920.4	4
**PF13353.9**	1473	PF06803.15	49	PF16976.8	14	PF14579.9	5
**PF13394.9**	1796	PF07314.14	9	PF01408.25	286	PF03692.18	7
**PF04055.24**	1821	PF09837.12	18	PF00190.25	30	PF19912.2	4
**PF13370.9**	4341	PF13556.9	18	PF13473.9	127	PF03130.19	1
**PF13459.9**	4366	PF09918.12	62	PF04126.16	76	PF11821.11	7
**PF06902.14**	1892	PF09947.12	10	PF18050.4	73	PF14557.9	3
**PF00210.27**	2320	PF10844.11	19	PF01928.24	218	PF13371.9	6
**PF02915.20**	2479	PF10763.12	23	PF02167.18	70	PF03330.21	7
**PF01883.22**	814	PF11984.11	4	PF09719.13	80	PF03291.19	3
**PF01325.22**	441	PF13081.9	15	PF07876.15	182	PF00127.23	4
**PF02742.18**	385	PF17898.4	22	PF01609.24	52	PF04085.17	1
**PF00254.31**	288	PF13487.9	12	PF13586.9	41	PF17645.4	3
**PF01613.21**	948	PF09551.13	70	PF02287.18	16	PF20095.2	8
**PF00258.28**	398	PF12105.11	26	PF01902.20	103	PF08714.14	5
**PF02525.20**	3494	PF01994.19	33	PF17124.8	184	PF14045.9	1

**PF03358.18**	4063	PF04794.15	51	PF13462.9	205	PF13435.9	5
**PF02441.22**	3350	PF07694.15	61	PF04077.15	127	PF04952.17	2
**PF02107.19**	157	PF17013.8	7	PF18357.4	64	PF11367.11	1
**PF05130.15**	434	PF01729.22	16	PF06866.14	93	PF13624.9	1
**PF00460.23**	5116	PF08821.14	27	PF06947.15	637	PF14472.9	1
**PF06429.16**	5885	PF08876.14	4	PF07853.14	92	PF01599.22	2
**PF02050.19**	163	PF09941.12	13	PF13630.9	91	PF19937.2	3
**PF03748.17**	426	PF09988.12	17	PF09911.12	67	PF16264.8	3
**PF02561.17**	390	PF04017.15	22	PF09969.12	27	PF03847.16	4
**PF02623.18**	675	PF14103.9	2	PF11256.11	145	PF10677.12	1
**PF04964.17**	7	PF06014.14	40	PF11385.11	108	PF05154.19	2
**PF04205.17**	281	PF01541.27	20	PF04241.18	363	PF04010.16	2
**PF02152.21**	3283	PF06560.14	9	PF04286.15	72	PF10622.12	5
**PF00551.22**	3130	PF01493.22	55	PF04306.16	199	PF01134.25	5
**PF05683.15**	1086	PF00126.30	9	PF18955.3	106	PF14606.9	3
**PF01475.22**	12042	PF01564.20	16	PF01207.20	215	PF01702.21	2
**PF02660.18**	3305	PF13679.9	17	PF08761.14	168	PF04182.15	4
**PF04309.15**	1153	PF03448.20	28	PF00889.22	750	PF01861.19	5
**PF07722.16**	10456	PF05402.15	14	PF01956.19	27	PF09892.12	2
**PF01174.22**	7880	PF01092.22	13	PF00756.23	180	PF18046.4	1
**PF03575.20**	1460	PF19489.2	3	PF02472.19	522	PF12673.10	3
**PF07670.17**	1845	PF17924.4	38	PF03807.20	425	PF19538.2	6
**PF01597.22**	7171	PF17939.4	28	PF01557.21	185	PF06940.14	1
**PF03009.20**	2358	PF00154.24	43	PF06490.14	39	PF09888.12	6
**PF00196.22**	310502	PF08423.14	36	PF00813.23	328	PF12669.10	1
**PF08281.15**	156882	PF08747.14	12	PF01728.22	468	PF05573.15	2
**PF04545.19**	30776	PF06778.15	153	PF12681.10	278	PF03843.16	1
**PF13384.9**	1924	PF00891.21	42	PF09338.14	58	PF12663.10	4
**PF13936.9**	787	PF00175.24	31	PF12464.11	296	PF12671.10	4
**PF16968.8**	193	PF07099.14	1	PF05015.16	79	PF13958.9	1
**PF02686.18**	13587	PF13025.9	8	PF08671.13	13	PF10387.12	1
**PF01182.23**	1358	PF14242.9	18	PF13413.9	105	PF03609.17	5
**PF00462.27**	1025	PF11611.11	10	PF09021.14	253	PF06160.15	2
**PF13192.9**	1080	PF04324.18	21	PF05116.16	51	PF01163.25	2
**PF00691.23**	343	PF17806.4	10	PF08282.15	133	PF06293.17	4
**PF13441.9**	67	PF03143.20	18	PF07885.19	7	PF09501.13	1
**PF13488.9**	75	PF01973.21	58	PF04362.17	173	PF02784.19	3

**PF00535.29**	1835	PF08239.14	31	PF02669.18	258	PF16256.8	4
**PF10111.12**	239	PF18348.4	8	PF03548.18	77	PF01627.26	3
**PF13641.9**	1172	PF13597.9	7	PF17131.7	14	PF03279.16	5
**PF03808.16**	605	PF14378.9	70	PF03788.17	34	PF06127.14	2
**PF00903.28**	5429	PF15781.8	15	PF05148.18	20	PF13650.9	1
**PF13468.9**	2213	PF16242.8	11	PF01106.20	733	PF13975.9	1
**PF13669.9**	4053	PF01191.22	21	PF08780.14	69	PF06984.16	1
**PF07702.16**	9958	PF08176.15	17	PF07009.14	246	PF04355.16	2
**PF12143.11**	626	PF02824.24	5	PF13505.9	87	PF04032.19	1
**PF13730.9**	70	PF12139.11	16	PF13568.9	78	PF04267.15	1
**PF14502.9**	380	PF00491.24	4	PF03479.18	96	PF07833.14	3
**PF07355.15**	51	PF04380.16	3	PF13798.9	13	PF04282.16	1
**PF01272.22**	8290	PF09209.14	12	PF07998.14	8	PF07291.14	1
**PF03449.18**	8277	PF11007.11	16	PF05135.16	30	PF08899.14	3
**PF01025.22**	102	PF06172.14	13	PF13396.9	34	PF09840.12	3
**PF00255.22**	964	PF07408.14	15	PF00874.23	11	PF15937.8	4
**PF00625.24**	2500	PF08680.13	36	PF00160.24	306	PF06005.15	1
**PF06888.15**	186	PF09846.12	17	PF01885.19	18	PF02326.18	1
**PF12710.10**	8087	PF09880.12	4	PF02245.19	474	PF13196.9	3
**PF00702.29**	13287	PF00013.32	13	PF14489.9	478	PF04344.16	1
**PF05822.15**	8	PF05597.14	16	PF03838.17	206	PF09967.12	1
**PF13419.9**	14096	PF04034.16	20	PF04607.20	66	PF09876.12	1
**PF13242.9**	9313	PF01868.19	5	PF01282.22	17	PF07663.14	4
**PF09419.13**	1014	PF01887.19	10	PF10099.12	7	PF10803.11	3
**PF01725.19**	6801	PF20257.1	10	PF13490.9	69	PF17261.5	7
**PF01966.25**	1615	PF03684.16	24	PF10263.12	195	PF02249.20	3
**PF08668.15**	39	PF03100.18	57	PF17283.5	143	PF08768.14	2
**PF13646.9**	14	PF04021.15	8	PF01336.28	24	PF07282.14	2
**PF01814.26**	912	PF02005.19	43	PF17935.4	82	PF05673.16	1
**PF01934.20**	460	PF01180.24	26	PF01974.20	19	PF07700.18	3
**PF17209.6**	329	PF06612.14	59	PF02778.17	19	PF05625.14	3
**PF00633.26**	1980	PF09001.14	13	PF05685.15	1267	PF14219.9	1
**PF10531.12**	510	PF09930.12	7	PF02699.18	172	PF06426.17	3
**PF12836.10**	503	PF12952.10	6	PF04307.17	54	PF06338.14	1
**PF00730.28**	1927	PF07505.14	27	PF01863.20	208	PF10634.12	1
**PF01330.24**	9408	PF13410.9	120	PF03734.17	78	PF14690.9	3
**PF07499.16**	8944	PF04138.17	41	PF09855.12	16	PF02274.20	1
**PF14520.9**	9439	PF13795.9	28	PF00931.25	91	PF01998.20	1
**PF15919.8**	241	PF03721.17	75	PF12730.10	267	PF12672.10	2

**PF01634.21**	2405	PF05336.16	113	PF18462.4	24	PF11181.11	7
**PF00977.24**	12550	PF01547.28	12	PF19571.2	314	PF19798.2	7
**PF01884.20**	182	PF13531.9	21	PF13911.9	67	PF03050.17	5
**PF00300.25**	6802	PF08970.13	25	PF01917.19	54	PF13817.9	5
**PF00403.29**	3939	PF08177.14	19	PF07441.14	96	PF17647.4	4
**PF01844.26**	166	PF08271.15	52	PF02361.19	39	PF13512.9	6
**PF13395.9**	80	PF10899.11	1	PF01903.20	168	PF13525.9	6
**PF14279.9**	150	PF00710.23	25	PF05732.14	3	PF01442.21	1
**PF10960.11**	9	PF07088.14	9	PF02633.17	90	PF04989.15	2
**PF01022.23**	2007	PF17656.4	19	PF08206.14	83	PF09893.12	4
**PF13463.9**	818	PF02596.18	25	PF04134.15	31	PF10842.11	1
**PF13601.9**	186	PF09879.12	7	PF02288.18	28	PF16316.8	7
**PF01527.23**	446	PF09963.12	28	PF09350.13	123	PF06249.15	2
**PF13518.9**	204	PF09365.13	35	PF17782.4	6	PF12692.10	2
**PF04542.17**	10595	PF11116.11	25	PF07085.15	195	PF13783.9	5
**PF01455.21**	164	PF14330.9	53	PF07796.14	20	PF14229.9	3
**PF01750.21**	284	PF08461.13	2	PF07849.14	32	PF17379.5	4
**PF08645.14**	92	PF01187.21	50	PF07870.14	472	PF19645.2	1
**PF01155.22**	2163	PF01927.19	29	PF08796.13	30	PF05489.15	1
**PF08858.13**	196	PF01931.21	34	PF02639.17	552	PF12811.10	1
**PF08864.13**	208	PF06769.17	31	PF08968.13	63	PF09990.12	1
**PF00707.25**	2261	PF00705.21	48	PF02681.17	327	PF02965.20	1
**PF05198.19**	2258	PF02747.18	29	PF10035.12	100	PF19741.2	4
**PF06135.15**	600	PF04139.16	21	PF09966.12	77	PF08448.13	1
**PF00857.23**	2301	PF04328.16	38	PF10031.12	17	PF04407.15	2
**PF01128.22**	5699	PF01026.24	6	PF10740.12	49	PF10049.12	1
**PF12804.10**	8117	PF09500.13	22	PF10750.12	10	PF08238.15	1
**PF01424.25**	2148	PF14005.9	24	PF11188.11	139	PF17926.4	2
**PF13083.9**	7258	PF14165.9	29	PF01796.20	43	PF11750.11	3
**PF14804.9**	2107	PF20077.2	3	PF12172.11	43	PF13045.9	3
**PF07689.15**	133	PF06961.16	17	PF13171.9	82	PF13612.9	3
**PF00189.23**	5791	PF13937.9	75	PF13566.9	101	PF14062.9	1
**PF07650.20**	5867	PF12728.10	17	PF14079.9	31	PF14514.9	4
**PF00467.32**	12334	PF01280.23	39	PF14221.9	65	PF16760.8	1
**PF17136.7**	10599	PF01194.20	18	PF01873.20	24	PF10080.12	9
**PF02357.22**	8961	PF00209.21	1	PF00071.25	18	PF01555.21	3
**PF02502.21**	8449	PF01988.22	13	PF13484.9	181	PF10727.12	2
**PF00753.30**	12680	PF02702.20	3	PF06289.14	121	PF10925.11	2
**PF12706.10**	8933	PF14908.9	70	PF04299.15	172	PF11497.11	5

**PF19583.2**	628	PF18289.4	58	PF01590.29	372	PF12732.10	2
**PF13483.9**	3003	PF06240.16	36	PF13185.9	540	PF07336.14	3
**PF04011.15**	2546	PF18750.4	7	PF13807.9	328	PF02656.18	1
**PF03588.17**	78	PF08713.14	67	PF00043.28	276	PF06080.15	4
**PF00717.26**	7275	PF02600.19	76	PF02798.23	306	PF02882.22	2
**PF01726.19**	6682	PF09882.12	3	PF13409.9	317	PF09932.12	9
**PF00657.25**	463	PF10732.12	20	PF13417.9	362	PF01655.21	6
**PF13472.9**	582	PF10884.11	1	PF13581.9	1333	PF14334.9	4
**PF01451.24**	4627	PF11518.11	3	PF03006.23	249	PF08921.14	3
**PF04172.19**	123	PF14066.9	26	PF01978.22	182	PF10112.12	3
**PF02589.18**	1028	PF14209.9	86	PF13022.9	5	PF14143.9	2
**PF00677.20**	5393	PF04256.15	2	PF01638.20	215	PF12147.11	1
**PF01810.21**	2871	PF18481.4	2	PF07486.15	508	PF01986.19	1
**PF04397.18**	2691	PF14326.9	15	PF03439.16	43	PF09055.14	1
**PF01661.24**	1319	PF19891.2	21	PF01790.21	39	PF06707.14	1
**PF02545.17**	5656	PF01346.21	28	PF02664.18	152	PF07589.14	1
**PF01575.22**	603	PF07972.14	32	PF06175.14	29	PF09505.13	2
**PF13452.9**	337	PF13513.9	4	PF01909.26	226	PF07653.20	1
**PF19315.2**	92	PF13011.9	49	PF18765.4	232	PF19586.2	2
**PF01914.20**	645	PF13565.9	49	PF11071.11	41	PF14819.9	1
**PF04014.21**	4422	PF13592.9	58	PF15891.8	7	PF14498.9	2
**PF15714.8**	3093	PF16584.8	19	PF03938.17	112	PF02237.20	4
**PF03819.20**	505	PF14582.9	33	PF02604.22	96	PF05258.15	1
**PF01502.21**	1131	PF15731.8	6	PF04351.16	34	PF02831.18	1
**PF01503.20**	1250	PF13366.9	22	PF08745.14	20	PF05534.15	3
**PF12643.10**	252	PF08478.13	44	PF12769.10	20	PF19024.3	2
**PF05389.15**	630	PF10584.12	7	PF13367.9	82	PF14597.9	2
**PF00376.26**	849	PF00181.26	46	PF03874.19	30	PF20420.1	1
**PF13411.9**	860	PF03947.21	46	PF04246.15	124	PF13921.9	1
**PF00149.31**	1566	PF02334.19	38	PF00101.23	29	PF14375.9	1
**PF12850.10**	3046	PF01947.19	6	PF03840.17	485	PF03927.16	5
**PF03141.19**	18	PF17937.4	7	PF19569.2	43	PF13715.9	2
**PF01135.22**	3096	PF09359.13	15	PF14278.9	274	PF07920.14	5
**PF07021.15**	488	PF14317.9	1	PF03459.20	9	PF08433.13	6
**PF03848.17**	319	PF14071.9	24	PF09976.12	173	PF13685.9	3
**PF01596.20**	2360	PF17774.4	9	PF02081.18	73	PF15738.8	3
**PF13578.9**	2401	PF06725.14	31	PF08868.13	253	PF10722.12	1
**PF04816.15**	927	PF09662.13	39	PF14183.9	26	PF05901.14	2
**PF12847.10**	1179	PF02969.20	56	PF09360.13	46	PF19187.3	3

**PF02384.19**	78	PF08006.14	49	PF01643.20	115	PF08807.13	1
**PF02390.20**	1574	PF10864.11	8	PF13346.9	195	PF13790.9	3
**PF04452.17**	3683	PF11419.11	1	PF06541.14	17	PF10816.11	1
**PF20260.1**	3478	PF11755.11	11	PF05368.16	819	PF12919.10	2
**PF02142.25**	959	PF04367.16	37	PF09527.13	59	PF01789.19	2
**PF02308.19**	966	PF05610.14	35	PF11599.11	5	PF09382.13	1
**PF03775.19**	812	PF04670.15	4	PF01027.23	85	PF14808.9	1
**PF05209.16**	378	PF12902.10	3	PF07362.15	2	PF04255.17	2
**PF03776.17**	308	PF01070.21	5	PF02962.18	34	PF05545.14	1
**PF00230.23**	2175	PF05690.17	26	PF02464.20	89	PF03969.19	2
**PF02659.18**	2076	PF07786.15	26	PF02627.23	246	PF06821.16	2
**PF05067.15**	980	PF00665.29	32	PF10672.12	69	PF10230.12	2
**PF01967.24**	5050	PF13276.9	9	PF02870.18	232	PF12389.11	2
**PF03205.17**	213	PF02613.18	21	PF10411.12	20	PF02006.19	4
**PF00994.27**	2044	PF01199.21	14	PF06335.15	104	PF13273.9	6
**PF04879.19**	161	PF01667.20	10	PF06570.14	23	PF17182.7	2
**PF03473.20**	529	PF08960.13	6	PF07301.14	22	PF10368.12	4
**PF03476.19**	104	PF02230.19	18	PF07659.14	2	PF12655.10	2
**PF01618.19**	688	PF13431.9	5	PF09878.12	9	PF00679.27	6
**PF02381.21**	7181	PF03264.17	9	PF10370.12	89	PF17299.5	5
**PF01741.21**	921	PF09875.12	4	PF10941.11	48	PF04955.15	1
**PF00893.22**	2896	PF18690.4	19	PF14004.9	53	PF02426.19	2
**PF02080.24**	10630	PF05751.14	35	PF16265.8	4	PF04314.16	3
**PF02254.21**	10621	PF06445.18	21	PF04703.15	23	PF09559.13	3
**PF03446.18**	1378	PF05168.17	16	PF03961.16	96	PF15780.8	2
**PF04131.17**	561	PF06305.14	7	PF00009.30	58	PF07303.16	1
**PF03892.17**	18	PF13036.9	8	PF04347.16	11	PF08963.13	3
**PF00334.22**	7429	PF17135.7	20	PF02663.17	153	PF07755.14	2
**PF01957.21**	252	PF09138.14	3	PF01227.25	2912	PF00155.24	1
**PF01592.19**	1920	PF00303.22	14	PF03031.21	33	PF01041.20	1
**PF00881.27**	4798	PF09680.13	18	PF12917.10	188	PF08915.14	1
**PF02579.20**	684	PF00115.23	29	PF13023.9	189	PF00553.22	1
**PF06941.15**	106	PF05899.15	57	PF04405.17	225	PF06276.15	2
**PF00483.26**	1132	PF07563.14	32	PF03746.19	126	PF19644.2	1
**PF00293.31**	3549	PF08850.14	15	PF04525.15	103	PF04659.16	3
**PF01029.21**	1632	PF10066.12	7	PF01423.25	29	PF10970.11	4
**PF05991.14**	1540	PF03799.18	20	PF13244.9	23	PF04098.18	1
**PF00486.31**	1443209	PF13248.9	36	PF20501.1	4	PF18665.4	2

**PF03709.18**	158	PF01322.23	24	PF01189.20	39	PF08955.13	3
**PF00215.27**	1778	PF08958.13	13	PF05494.15	84	PF05405.17	3
**PF00213.21**	3898	PF09928.12	26	PF13677.9	239	PF09884.12	2
**PF02566.22**	813	PF18446.4	12	PF01402.24	305	PF10698.12	2
**PF00174.22**	110	PF01287.23	22	PF08753.14	202	PF00970.27	1
**PF00420.27**	4130	PF06155.15	2	PF14803.9	12	PF00248.24	1
**PF00499.23**	586	PF13542.9	39	PF14815.9	138	PF12718.10	1
**PF00507.22**	4099	PF03550.17	19	PF01936.21	13	PF20457.1	3
**PF00543.25**	20950	PF03169.18	2	PF10400.12	72	PF03682.16	1
**PF01507.22**	481	PF06810.14	15	PF05016.18	361	PF09472.13	1
**PF01161.23**	184	PF01157.21	27	PF07352.15	7	PF01954.19	1
**PF00398.23**	311	PF01198.22	30	PF05137.16	60	PF18556.4	2
**PF02452.20**	4356	PF05857.14	32	PF00484.22	333	PF18946.3	1
**PF03965.19**	240	PF00089.29	2	PF04024.15	163	PF05275.14	1
**PF01252.21**	840	PF13365.9	2	PF00239.24	230	PF19174.3	3
**PF01470.20**	888	PF08889.14	46	PF01694.25	295	PF07295.14	1
**PF00557.27**	5137	PF14007.9	19	PF00352.24	64	PF14537.9	2
**PF04327.15**	2065	PF07524.16	15	PF03070.19	106	PF12910.10	1
**PF10502.12**	2984	PF16036.8	16	PF02909.20	114	PF10069.12	1
**PF01195.22**	11630	PF01972.19	13	PF03966.19	214	PF03612.17	1
**PF01327.24**	2934	PF10414.12	14	PF03350.19	106	PF11554.11	1
**PF04020.16**	756	PF14285.9	5	PF14003.9	35	PF01946.20	1
**PF00589.25**	196	PF02662.19	39	PF06908.14	94	PF14137.9	1
**PF08890.14**	73	PF01551.25	19	PF14038.9	33	PF08222.14	2
**PF01895.22**	10756	PF00832.23	34	PF06983.16	147	PF13751.9	1
**PF01865.19**	2696	PF00872.21	13	PF01118.27	24	PF14715.9	1
**PF02811.22**	954	PF12637.10	35	PF00004.32	37	PF13197.9	1
**PF02585.20**	528	PF07015.14	11	PF00308.21	299	PF07452.15	1
**PF07238.17**	558	PF03029.20	14	PF18277.4	98	PF03691.17	1
**PF12945.10**	495	PF08477.16	7	PF04548.19	87	PF15980.8	1
**PF01625.24**	275	PF01148.23	18	PF03773.16	174	PF01734.25	1
**PF01243.23**	418	PF01864.20	32	PF04266.17	6	PF04636.16	1
**PF10590.12**	219	PF02578.18	25	PF17805.4	151	PF20617.1	2
**PF12766.10**	41	PF07905.14	7	PF13549.9	1	PF02136.23	1
**PF01048.23**	4485	PF07843.14	20	PF01869.23	13	PF12680.10	2
**PF03364.23**	370	PF03266.18	24	PF00188.29	75	PF06999.15	1
**PF10604.12**	462	PF10649.12	6	PF01693.19	15	PF12666.10	2
**PF01522.24**	243	PF10830.11	13	PF15630.9	45	PF01845.20	1
**PF01558.21**	4735	PF14063.9	10	PF04218.16	18	PF13590.9	2

**PF00550.28**	3678	PF06161.14	23	PF10628.12	97	PF19027.3	2
**PF14573.9**	265	PF10639.12	15	PF04977.18	123	PF15619.9	2
**PF00481.24**	3464	PF12019.11	29	PF07261.14	148	PF16431.8	2
**PF07228.15**	2178	PF13298.9	9	PF02481.18	4	PF00083.27	1
**PF13672.9**	4129	PF10125.12	11	PF07681.15	79	PF07690.19	1
**PF00697.25**	3505	PF05951.16	7	PF02001.19	108	PF12832.10	1
**PF01782.21**	8914	PF08291.14	7	PF07293.14	138	PF13347.9	1
**PF05239.19**	8999	PF00866.21	6	PF07556.14	98	PF09845.12	1
**PF01255.22**	1163	PF01193.27	4	PF09889.12	4	PF16745.8	1
**PF14681.9**	15446	PF13656.9	11	PF10702.12	5	PF01246.23	3
**PF14464.9**	974	PF17922.4	79	PF11023.11	34	PF10771.12	1
**PF00227.29**	6283	PF04366.15	4	PF03860.19	10	PF00261.23	1
**PF11563.11**	15	PF18174.4	6	PF12225.11	84	PF05266.17	1
**PF01416.23**	3736	PF01610.20	5	PF19601.2	62	PF05911.14	1
**PF00849.25**	20121	PF13852.9	4	PF19810.2	2	PF06008.17	1
**PF01479.28**	41620	PF14344.9	5	PF06149.15	211	PF06818.18	1
**PF04012.15**	675	PF19618.2	5	PF05653.17	22	PF16729.8	2
**PF06130.15**	374	PF02240.19	2	PF01996.19	12	PF00291.28	1
**PF01242.22**	1042	PF00069.28	4	PF04060.16	74	PF13444.9	3
**PF00381.22**	6443	PF12900.10	38	PF00498.29	221	PF04193.17	2
**PF03829.16**	179	PF01020.20	12	PF12401.11	40	PF04365.16	1
**PF03830.18**	1406	PF16295.8	23	PF16697.8	221	PF07866.14	1
**PF00358.23**	741	PF03073.18	19	PF12641.10	146	PF18573.4	2
**PF00359.25**	4592	PF03683.16	10	PF04961.15	622	PF14147.9	1
**PF00367.23**	71	PF14864.9	3	PF13653.9	151	PF14478.9	2
**PF02255.19**	607	PF11724.11	18	PF01184.22	5	PF20126.2	1
**PF02302.20**	4323	PF10237.12	7	PF02518.29	1497	PF20458.1	2
**PF02700.17**	2278	PF03232.16	14	PF07927.15	98	PF14501.9	1
**PF00719.22**	477	PF14709.10	22	PF04883.15	41	PF02089.18	2
**PF02568.17**	84	PF11009.11	55	PF07456.14	141	PF03496.17	1
**PF06508.16**	1625	PF13031.9	20	PF03641.17	113	PF13893.9	3
**PF02677.17**	132	PF13711.9	53	PF13591.9	10	PF01636.26	1
**PF06177.14**	433	PF14332.9	23	PF09936.12	43	PF19974.2	2
**PF04002.18**	4221	PF20122.2	9	PF03060.18	228	PF18478.4	2
**PF20582.1**	4054	PF04313.17	2	PF01784.21	12	PF16917.8	1
**PF02033.21**	1471	PF13588.9	24	PF02501.20	14	PF10551.12	1
**PF05025.16**	1635	PF18546.4	7	PF16552.8	51	PF19480.2	1
**PF02132.18**	17427	PF01015.21	9	PF06114.16	45	PF19502.2	1
**PF13662.9**	17871	PF14069.9	39	PF10076.12	27	PF04410.17	2

**PF02631.19**	263	PF01253.25	26	PF13470.9	110	PF16921.8	2
**PF01042.24**	10651	PF10025.12	11	PF02563.19	53	PF04705.15	1
**PF14588.9**	2022	PF11576.11	6	PF01200.21	18	PF12570.11	3
**PF00687.24**	16570	PF00290.23	4	PF01201.25	51	PF00454.30	2
**PF00466.23**	7810	PF07022.16	2	PF04308.15	297	PF18846.4	1
**PF00298.22**	19859	PF06296.15	2	PF03259.20	107	PF07497.15	1
**PF03946.17**	19847	PF14006.9	21	PF14361.9	11	PF03144.28	6
**PF00542.22**	17799	PF09860.12	30	PF05866.14	17	PF10826.11	1
**PF16320.8**	17798	PF10949.11	11	PF02630.17	179	PF02550.18	1
**PF00572.21**	14304	PF11010.11	11	PF02810.18	87	PF19300.2	1
**PF00238.22**	20294	PF04019.15	14	PF03695.16	50	PF18854.4	1
**PF00252.21**	16046	PF16789.8	16	PF01641.21	303	PF13335.9	1
**PF00861.25**	6548	PF09684.13	2	PF04079.19	198	PF13343.9	1
**PF01245.23**	10025	PF01411.22	16	PF04203.16	80	PF14172.9	1
**PF00453.21**	13373	PF07973.17	15	PF03567.17	19	PF13103.9	1
**PF00829.24**	14244	PF10087.12	21	PF04612.15	31	PF09877.12	2
**PF00237.22**	7442	PF11325.11	12	PF02416.19	99	PF11483.11	1
**PF01386.22**	1674	PF13464.9	31	PF05099.16	24	PF20208.1	1
**PF14693.9**	1674	PF04250.16	34	PF17754.4	57	PF05233.16	1
**PF01016.22**	4820	PF17253.5	24	PF01096.21	50	PF07879.14	1
**PF00828.22**	15067	PF03065.18	10	PF14451.9	223	PF12276.11	1
**PF00830.22**	10606	PF12826.10	37	PF02371.19	10	PF07298.14	1
**PF00831.26**	1986	PF05891.15	12	PF02588.18	31	PF17294.5	1
**PF00297.25**	16473	PF13190.9	11	PF14036.9	18	PF16575.8	1
**PF00327.23**	5675	PF04717.15	3	PF13717.9	7	PF04237.16	1
**PF01197.21**	4644	PF01855.22	5	PF02826.22	164	PF11141.11	1
**PF01783.26**	6035	PF02776.21	17	PF06745.16	220	PF16732.8	1
**PF00471.23**	8935	PF14141.9	38	PF13481.9	109	PF07606.14	1
**PF00468.20**	6757	PF14174.9	10	PF03992.19	126	PF05719.14	1
**PF01632.22**	13983	PF03767.17	19	PF13336.9	115	PF09509.13	1
**PF00573.25**	9424	PF09881.12	3	PF14542.9	87	PF00950.20	1
**PF00935.22**	48	PF13047.9	49	PF06857.14	39	PF01032.21	1
**PF00281.22**	16507	PF04854.17	21	PF00107.29	39	PF05523.14	1
**PF00673.24**	16507	PF02433.18	5	PF06094.15	71	PF06082.14	3
**PF00347.26**	19582	PF03597.18	6	PF13772.9	34	PF01548.20	3
**PF01248.29**	1371	PF00078.30	1	PF01871.20	84	PF09537.13	1
**PF01281.22**	16675	PF08388.14	1	PF08280.14	37	PF07666.14	1
**PF03948.17**	16675	PF08797.14	2	PF07100.14	92	PF02867.18	1
**PF00338.25**	15679	PF13100.9	1	PF06180.14	40	PF14064.9	1

**PF00411.22**	8274	PF04066.16	35	PF01339.20	30	PF09929.12	1
**PF00416.25**	17611	PF04210.16	3	PF20229.1	29	PF03083.19	1
**PF00253.24**	14668	PF10126.12	8	PF00795.25	36	PF12441.11	1
**PF00312.25**	14792	PF02150.19	35	PF14522.9	27	PF06252.15	1
**PF00886.22**	2685	PF04259.17	3	PF00034.24	299	PF03351.20	1
**PF00366.23**	3683	PF07812.15	3	PF00926.22	15	PF09459.13	1
**PF01084.23**	1377	PF07374.14	8	PF07719.20	43	PF07704.14	2
**PF00203.24**	11713	PF12007.11	31	PF06569.14	210	PF00395.23	2
**PF01165.23**	2975	PF01183.23	8	PF08897.14	43	PF06291.14	2
**PF02482.22**	7313	PF12323.11	9	PF09954.12	47	PF04945.16	1
**PF16321.8**	7271	PF13344.9	14	PF09986.12	231	PF18681.4	1
**PF00163.22**	20930	PF06835.16	8	PF10652.12	38	PF09388.13	1
**PF00333.23**	3967	PF04630.15	7	PF10942.11	77	PF10142.12	1
**PF03719.18**	3967	PF01775.20	10	PF11117.11	185	PF13618.9	1
**PF01250.20**	9591	PF08175.15	26	PF11127.11	126	PF02411.18	1
**PF00177.24**	19361	PF04536.17	2	PF11667.11	4	PF03190.18	1
**PF00410.22**	20255	PF08325.13	40	PF04027.16	15	PF00685.30	1
**PF00380.22**	7413	PF14151.9	28	PF04260.15	458	PF10447.12	1
**PF00164.28**	13048	PF01738.21	35	PF14584.9	130	PF13377.9	1
**PF00834.22**	12004	PF11859.11	20	PF04379.17	258	PF13577.9	1
**PF00355.29**	873	PF14272.9	4	PF19799.2	4	PF07991.15	2
**PF13806.9**	354	PF02925.19	4	PF01940.19	13	PF17925.4	1
**PF10399.12**	332	PF03054.19	15	PF06107.14	227	PF20498.1	1
**PF00075.27**	852	PF02922.21	2	PF02661.21	114	PF06170.15	1
**PF13456.9**	116	PF01817.24	2	PF12611.11	95	PF19668.2	1
**PF01351.21**	250	PF11582.11	18	PF08704.13	94	PF19851.2	1
**PF01138.24**	2298	PF20398.1	10	PF13589.9	279	PF17648.4	1
**PF03725.18**	2252	PF01912.21	39	PF07432.16	19	PF05636.14	1
**PF00929.27**	493	PF01794.22	6	PF06787.14	4	PF14148.9	1
**PF16473.8**	104	PF03444.18	11	PF02602.18	5	PF13640.9	1
**PF02508.17**	1639	PF09123.14	27	PF17836.4	509	PF13661.9	1
**PF00639.24**	41	PF10710.12	7	PF01418.20	58	PF07498.15	1
**PF13145.9**	41	PF13450.9	14	PF02002.20	11	PF00700.24	1
**PF13616.9**	41	PF05049.16	8	PF01899.19	86	PF01116.23	1
**PF07288.14**	166	PF13328.9	28	PF02391.20	122	PF07578.14	1
**PF03737.18**	304	PF05958.14	81	PF08378.14	140	PF00136.24	2
**PF01765.22**	17362	PF15898.8	13	PF06042.14	17	PF10061.12	1
**PF02082.23**	5093	PF11139.11	8	PF11612.11	54	PF13280.9	1
**PF06962.15**	1010	PF04173.16	11	PF04277.16	11	PF14139.9	1

**PF02410.18**	908	PF08901.14	31	PF02678.19	184	PF05656.17	1

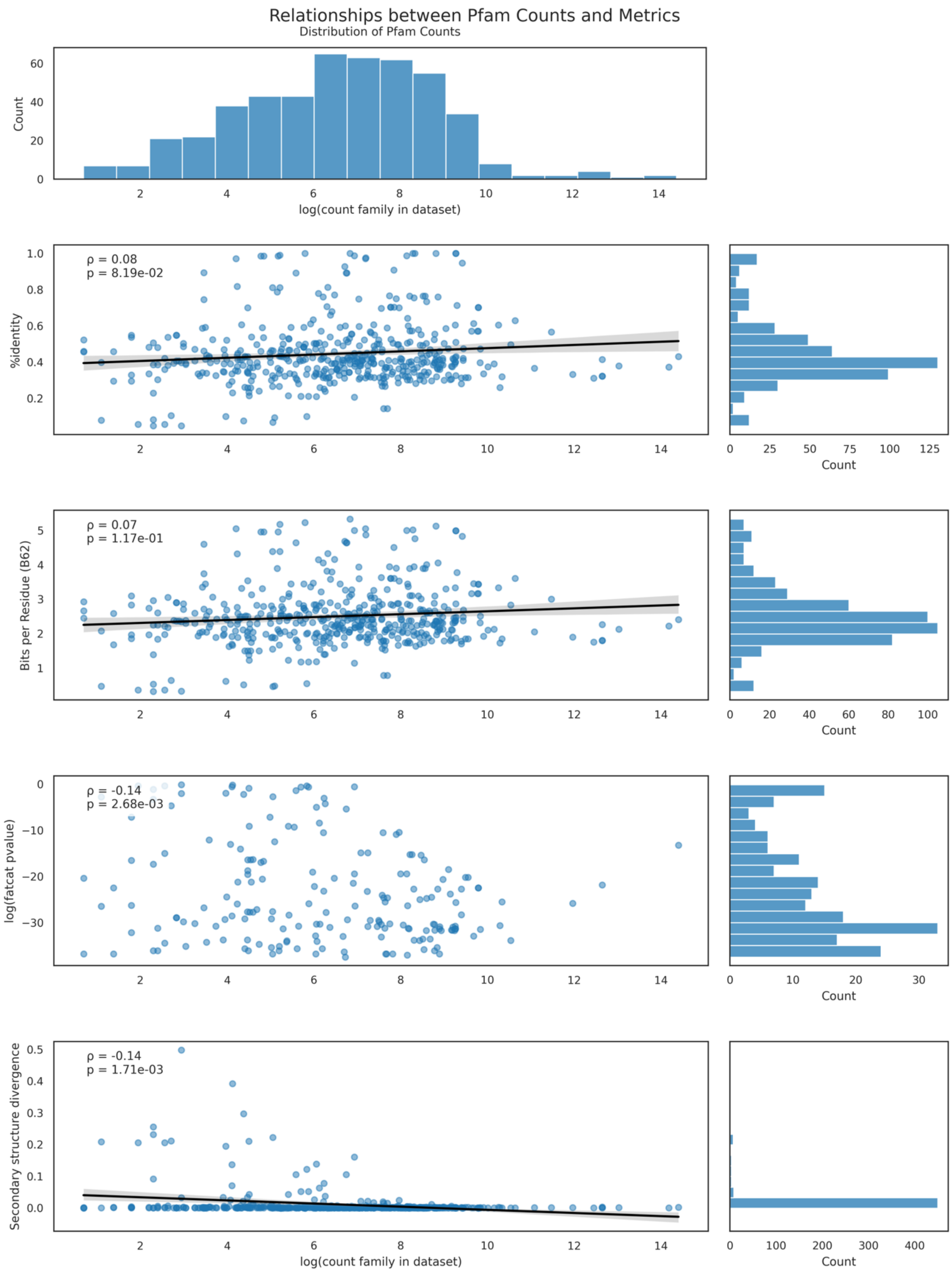

Figure S3: Test set metrics on a per family basis for the examples in the test set with Pfam labels (479) against the number of times the family is found in the dataset.[2] We observe a tendency for decreased performance as the family becomes more "rare" to the model. In some cases, the

BEAM search collapses and the model does not create a protein that matches the same predicted fold as the thermophilic homolog (log FATCAT >-3).[3]

Alignment of NOMELT EnHD Variant to Wild Type

```
DKRPRTAFSSEQLARLKREFNENRYLTERRRQQLSSELGLNEAQIKIWFQNKRAKIKK-----
.|||||||..|||.|||.||...|||||.|||.|..||||||.|||||||||||||||
EKRPRTAFTAEQLQRLKAEFQASRYLTEQRRQELARELGLNESQIKIWFQNKRAKIKKASGNK
```

Figure S4: Alignment of wild type EnHD (top) to NOMELT generated variant (bottom).

Creating a second “optimized” EnHD variant

In addition to taking the raw 14 mutation output of NOMELT, we viewed it as a generator of variation, given that the model may be introducing variation picked up from nature that does not directly impact thermal stability. This yields a library size of 16,384 considering 14 binary mutations to search over.

We conducted 10 rounds of NSGA-ii evolutionary optimization each with a population size of 10 using the mAF-min method as an objective function. These rounds explore only 0.6% of the library, but are able to improve the estimated stability score of EnHD from 2.37 to to 2.51 using NOMELT suggestions. We then repeated this process considering 14 random mutations as opposed to the 14 suggested by NOMELT, and were unable to significantly improve score. See Figure S1. The optimized variant did non however exhibit an increase in stability as measured by MD or experimentally.

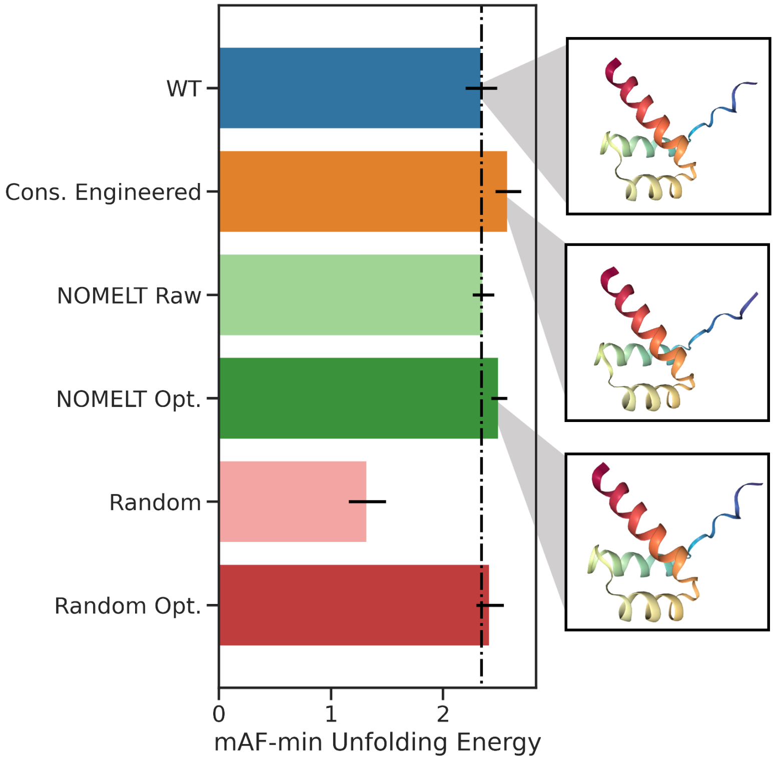

Figure S5 Comparison of stability of various designs according to the mAF-min method's unfolding free energy change. Error bars indicate 95% CI over the ensemble of AlphaFold structures.[4] Vertical black line is the wild type's score. The previously engineered variant, by consensus over many homologs (orange) is more stable according to the estimator (P-value $1.7e^{-19}$). The raw output of the NOMELT model given a single input wild type sequence (light green), which includes 14 mutations involving some insertions, is not stabilizing or destabilizing. After only 100 examples from the search space of 14^2 mutation permutations have been explored using NSGA-ii (dark green), we achieve a statistically more stable variant than WT (P-value $9.5e^{-14}$). Introducing the same number of mutations randomly is extremely destabilizing (light red), and running the same number of exploration steps over the random search space cannot improve the protein over wild type.

1. Komp, E. *et al.* Homologous Pairs of Low and High Temperature Originating Proteins Spanning the Known Prokaryotic Universe. *Sci. Data* **10**, 682 (2023).
2. Mistry, J. *et al.* Pfam: The protein families database in 2021. *Nucleic Acids Res.* **49**, D412–D419 (2021).
3. Li, Z., Jaroszewski, L., Iyer, M., Sedova, M. & Godzik, A. FATCAT 2.0: towards a better understanding of the structural diversity of proteins. *Nucleic Acids Res.* **48**, W60–W64 (2020).
4. Peccati, F., Alunno-Rufini, S. & Jiménez-Osés, G. Accurate Prediction of Enzyme Thermostabilization with Rosetta Using AlphaFold Ensembles. *J. Chem. Inf. Model.* **63**, 898–909 (2023).
